# Determinants of condom use among sexually active youth in SBC intervention communities in Tanzania: A cross-sectional study

**DOI:** 10.1371/journal.pone.0326878

**Published:** 2025-07-02

**Authors:** Mark Lwakatare, Gretchen Thompson, Joseph Msofe, Waziri Nyoni, Claire Gillum, Kara Tureski

**Affiliations:** 1 Family Health International (FHI 360), Dar es Salaam, United Republic of Tanzania; 2 Family Health International (FHI 360), Durham, North Carolina, United States of America; 3 Johns Hopkins Center for Communication Programs (CCP), Dar es Salaam, United Republic of Tanzania; 4 Family Health International (FHI 360), Washington D.C, United States of America; Makere University School of Public Health, UGANDA

## Abstract

HIV and unplanned pregnancies are significant public health concerns for youth in Sub-Saharan Africa. Consistent condom use is an effective behavioral method in preventing both health outcomes; however, it remains low and is declining among this population. Evidence indicates that preventive health behaviors are influenced by a range of specific factors relevant to their contexts. This study aimed to assess factors associated with condom use among sexually active youth in communities where social and behavior change (SBC) interventions were implemented in Tanzania. A cross-sectional study was conducted among 1,959 youths aged 15–24 years, selected using a multi-stage sampling approach from three regions (Iringa, Mwanza, and Tabora) where SBC interventions were implemented. Of these, 1,265 participants (64.6%) reported sexual activity within the six months preceding the study and were included in the analysis. Multivariable logistic regression was employed to determine the significant factors associated with condom use at last sex. *P*-values of ≤ 0.05 were considered statistically significant. Overall, 46.9% (95% CI: 44.0–50.0) of sexually active youth reported condom use at last sex, with higher prevalences observed among those exposed to condom use messaging through social media (70.4%), and who discussed condom use with their sexual partners (63.6%) and parents (62.2%). Significant predictors of condom use included residing in Tabora (AOR = 1.609; 95% CI: 1.155–2.241), having no children (AOR = 1.844; 95% CI: 1.095–3.106), delayed sexual debut (AOR = 1.591; 95% CI: 1.027–2.464), having multiple sexual partners (AOR = 1.667; 95% CI: 1.280–2.171), having a partner tested for HIV (AOR = 1.671; 95% CI: 1.138–2.455), self-efficacy to convince partner on condom use (AOR = 1.653; 95% CI: 1.233–2.215), discussing condom use with parents (AOR = 1.902; 95% CI: 1.430–2.532), and perceived parental support in HIV prevention (AOR = 1.585; 95% CI: 1.200–2.095). Condom use among sexually active youth in Tanzania is influenced by a range of sociodemographic, behavioral, and ideational (emotional and social) factors. This indicates the continued need for tailored multi-level interventions that address the contextual needs of target audiences to improve condom use. Further research is also needed to examine the causal pathways and underlying factors influencing condom use among sexually active youth.

## Introduction

Since the first detection of HIV more than 40 years ago, millions of infections and AIDS-related deaths have been prevented and reduced through new diagnostics, prevention tools, and treatment regimens. However, the world remains off track to meet the global goal of ending AIDS by 2030 and achieving the UNAIDS 2025 targets, particularly regarding knowledge of HIV status, despite these progressive efforts [1]. Additionally, progress toward these targets varies significantly by geography, age, and gender. There has been an overall increase in the number of new HIV infections and AIDS-related deaths in Eastern Europe and Central Asia compared to Sub-Saharan Africa, Asia, the Pacific, and the Caribbean, where there has been a notable reduction in new infections [2]. In Sub-Saharan Africa, new HIV infections continue to be disproportionately high among children, adolescent girls, and young women. In Tanzania, the prevalence of HIV among females aged 15–24 years (2.1%) is more than double that of males in the same age group (0.6%), and fewer than half of female youth with HIV are aware of their status [3]. The age of sexual debut for adolescents in Tanzania is as low as 9 years, which further exposes youth to a higher risk of acquiring HIV and other sexually transmitted infections (STIs) [4].

Unintended pregnancy remains another significant public health challenge in both developed and developing countries. Nearly half of all pregnancies, totaling 121 million each year globally, are unintended, and 61% of these unintended pregnancies end in induced abortion [5]. Furthermore, unplanned pregnancies occur frequently among younger populations. Annually, 12 million girls aged 15–19 years give birth, while 777,000 births occur among adolescent girls below 15 years in developing countries [6,7]. In Tanzania, 27% of teenage girls aged 15–19 are already mothers or are pregnant with their first child, which increased from 26% in 2005 to 23% in 2010 [8]. The factors that contribute to adolescent pregnancies include pressure for girls to marry and bear children early, limited educational and employment prospects, a lack of autonomy in health decisions, barriers to contraception access, health provider bias, sexual violence, and coercion [9]. Early and unintended pregnancies can lead to adverse health, educational, social, and economic consequences for adolescents. Pregnancy and childbirth complications are the leading cause of death among girls aged 15–19 years globally; unmarried pregnant adolescents encounter social stigma, rejection, or violence by partners, parents, and peers; adolescent pregnancy and childbearing often lead girls to drop out of school, which in turn affects their educational and employment opportunities [10].

Several efforts have been made to address these public health challenges. Still, condom use remains the most effective behavioral method for preventing HIV and unplanned pregnancies among sexually active populations when adopted correctly and consistently [11]. Since 1990, about 117 million new HIV infections have been prevented globally, while the unintended pregnancy rate has also declined from 79 to 64 per 1,000 women of reproductive age because of increased condom use [12,13]. However, global condom use has declined in recent years among those aged 15 and above, impeding progress toward the global 90% condom use target [14]. This is evident in Sub-Saharan Africa, where it is estimated that 60% of adolescents do not use condoms [15]. In Tanzania, the prevalence of condom use at last sex among youth aged 15–24 years has declined from 32.0% in 2016 to 26.1% in 2023 [3,16]. Therefore, these declining trends underscore the need to further understand the factors influencing condom use behavior among sexually active youth in an effort to contribute toward achieving global health targets [4,16].

Studies have indicated that many health behaviors are influenced by a variety of specific factors or determinants rather than by individual determinants alone. Preventive health behaviors are shaped by fear of disease, concerns about the cost or inconvenience of protective measures, confidence or doubts regarding the effectiveness of a treatment, and the motivation to follow the actions of others in the community [17]. In Tanzania, several studies have assessed various factors associated with condom use among youth [18–20]. Katikiro et al. found that feelings of shyness when purchasing condoms, perceptions that condoms reduce pleasure, perceived susceptibility and severity of HIV, experiences of forced sex, and the inability to persuade a partner to use condoms were predictors of non-condom use among out-of-school youths in Dar es Salaam [18]. Njau et al. revealed that age, religion, multiple sexual partners, and perceived barriers were determinants of condom use among secondary school students in Mbeya [19]. Kalolo et al. found that empowerment and a positive attitude toward condom use were predictors of condom use among sexually active adolescents in Mtwara [20].

Despite existing evidence on youth sexual behavior, there is limited research on the factors associated with condom use among sexually active youth within intervention-targeted communities, regardless of direct exposure to these interventions. Understanding behavioral patterns along with intervention exposure, psychosocial, and socio-demographic factors in such settings is crucial to inform effective programming. Therefore, this study aimed to assess the factors associated with condom use among sexually active youth in communities where social and behavior change (SBC) interventions were implemented in Tanzania. By focusing on intervention communities, the study aimed to reflect the real-world dynamics of behavior change in settings targeted by SBC efforts, while also assessing whether exposure was associated with the reported behavior. Given the high risk of HIV and unplanned pregnancies among youth, the findings from this study are essential for informing effective, context-specific strategies to improve sexual and reproductive health outcomes.

Our study is guided by a conceptual framework adapted from the Ideation Model of Strategic Communication and Behavior Change, which serves as the theoretical foundation for understanding the factors associated with condom use behavior among sexually active youth in Tanzania. This model is part of a metatheory of strategic communication and behavior change that hypothesizes psychosocial factors have a direct, complementary, and cumulative influence on health behavior change [17]. These psychosocial factors are grouped into three main domains or constructs, namely:

(a) * Cognitive determinants* of behavior, such as knowledge, belief, attitudes toward and perceived norms about the recommended behavior, perceived risk (of infection), and self-efficacy to protect oneself and/or others;
(b) * Emotional determinants*, such as feelings of fear, trust, confidence, self-efficacy, and empathy; and
(c) * Social influence determinants*, such as social support to practice the recommended behavior or peer pressure to avoid it and interpersonal communication with others about recommended practices [17,21].

The model further stipulates that these factors can be influenced by environmental context, social interaction, and exposure to different forms of communication (instruction, directive, non-directive, public), which increases the likelihood of population-level change [21,22]. This model has been used in previous studies to identify a variety of factors that predict health behavior or attribute behavior change to communication interventions in reproductive health, HIV and AIDS, Malaria, and Ebola [17,21].

**Fig 1:**
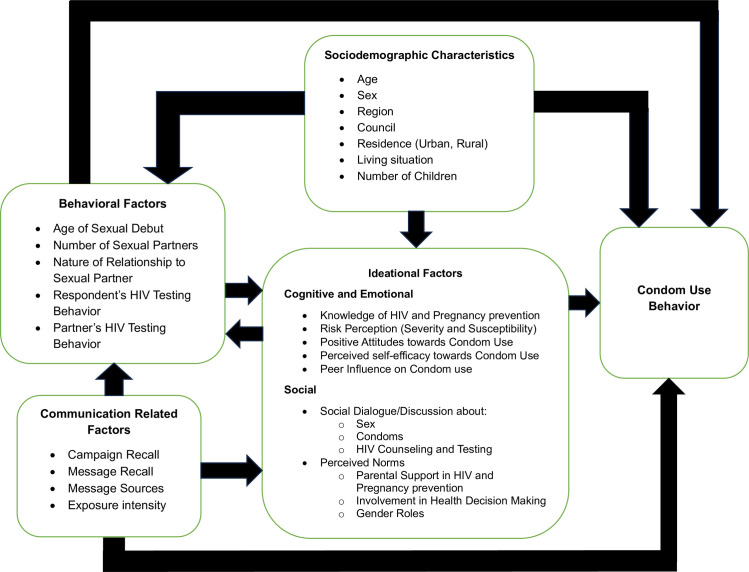
Conceptual framework.

Using the conceptual framework (Fig 1) below, this study aimed to assess factors associated with condom use among sexually active youth in communities where social and behavior change (SBC) interventions were implemented. These factors included communication-related variables (exposure, recall of campaign and messaging, source of exposure, and intensity of exposure), ideational factors (knowledge, risk perception, social dialogue, positive attitudes, perceived self-efficacy, and perceived norms), sociodemographic characteristics (age, sex, region, council, residence, living situation, and number of children), and behavioral factors (age of sexual debut, number of sexual partners, nature of relationship to sex partner, and HIV testing behavior).

## Materials and methods

### Study design

This study employed a cross-sectional design to test the conceptual framework related to condom use among sexually active youth. The design was chosen to examine the relationships between theoretical determinants of behavior and reported condom use, capturing insights into how these variables interact [23]. Quantitative data were collected to evaluate causal hypotheses derived from the theoretical framework and to evaluate the overall fit of the model to the observed data [24]. The research design also enabled to assess the strength of associations between variables specified in the tested hypotheses, supporting a rigorous evaluation of the conceptual framework.

### Study setting

This study was conducted in three regions of Tanzania—Iringa, Mwanza, and Tabora—as part of the Tulonge Afya project, a five-year (2017–2022) integrated social and behavior change (SBC) initiative funded by the United States Agency for International Development (USAID). The project aimed to improve health outcomes by addressing socio-cultural norms and promoting the adoption of healthy behaviors [25]. Implemented in 29 councils across 12 regions, Tulonge Afya used a multi-level approach that combined national mass media, social media, and community level activities.

To address the sexual and reproductive health needs of youth, the project launched the SITETEREKI (“unshakeable” in english) platform. This SBC platform promoted key behaviors including condom use, modern contraceptive uptake, HIV testing and treatment adherence, and voluntary medical male circumcision (VMMC). Messaging was delivered through a combination of mass media activities (radio spots, presenter interactions, interactive radio programs), social media (digital content), mid media activities (public address systems, cultural theatre, road shows), print media (posters, billboards, brochures, comic books), and interpersonal communication activities (small group dialogues facilitated by trained peer champions). These efforts targeted individual, social, and structural determinants of health behavior among young people.

### Population of the study

The study population included unmarried male and female youth aged 15–24 years living in communities where integrated SBC interventions were implemented. Participants were recruited from six intervention councils within the three selected regions of the Tulonge Afya project to assess patterns of behavior and factors associated with condom use in these settings. They were included because they were likely to be directly or indirectly influenced by the broader context of SBC activities implementation.

We excluded pregnant individuals, those who were too ill to participate in an interview, as well as peer educators or champions, community volunteers, and health workers to minimize potential bias related to professional or programmatic involvement in health promotion activities.

### Sample size and sampling

**Fig 2:**
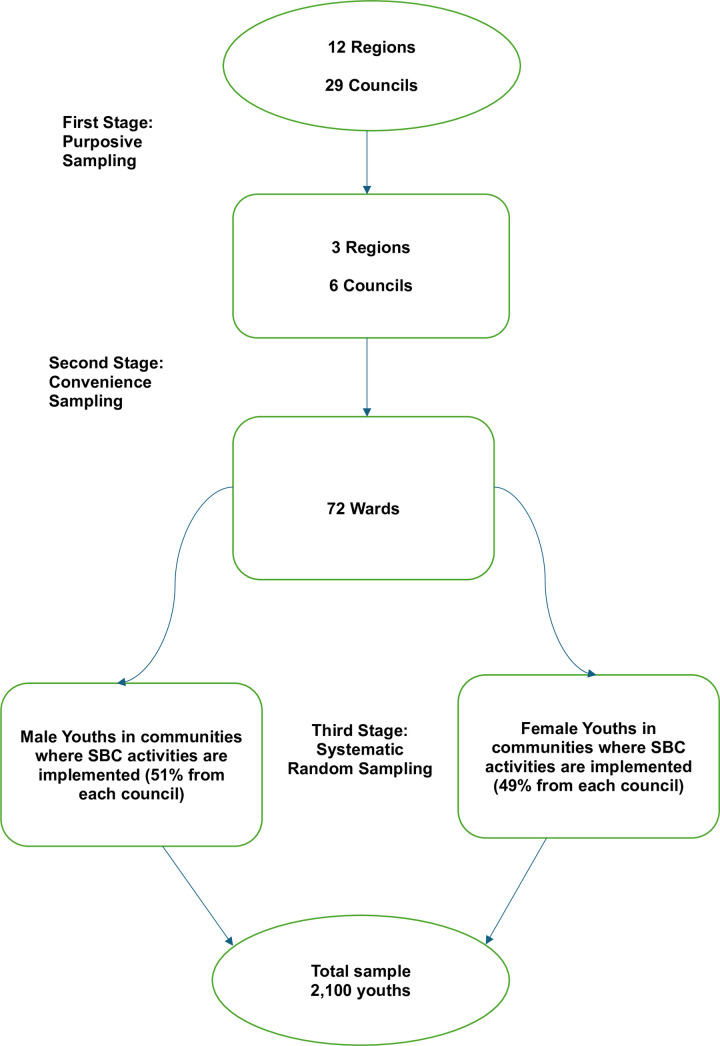
Step-by-step multistage sampling procedure.

The sample size determination considered the multistage sampling approach used (Fig 2). We employed a design effect of 2 to detect a minimum change of 5% from the condom use estimates in Tanzania, with a power of 80% for two-sided comparisons at a 5% significance level, resulting in a sample size of 2,400. However, the study could not accommodate larger sample sizes due to budget limitations and the resources needed for data collection and analysis. Therefore, a sample size of 2,100 respondents was selected for the study across the chosen regions and councils. A multi-stage sampling approach involving both non-probability and probability techniques was utilized. First, the selection of study sites (regions and councils) was purposive, based on varying contexts, SBC implementation, and the prevalence of HIV, teenage pregnancy, and modern contraception use [3,8]. Second, the selection of wards within those councils was convenient, based on ease of access (short-distance travel). Third, the study respondents were selected through systematic random sampling from separate lists (sampling frame) of male and female youths in communities where the SBC project activities were implemented. This sampling frame was obtained from the Tulonge Afya project database, which included the names, ages, genders, phone numbers, and locations (ward, council, region) of these youths. An equal allocation of the sample size across the selected regions was made from the total sample size to achieve representativeness.

### Data collection procedures and methods

Data was collected in August 2021 across six councils within the three selected regions. A team of 40 trained data collectors, fluent in both Kiswahili and English and experienced in quantitative research in Tanzania, implemented the fieldwork. All data collectors completed a three-day training that covered the study protocol, standard operating procedures, ethical considerations, and use of the data collection tools. Additionally, all team members completed an accredited human subjects research ethics training prior to data collection. In each council, at least seven data collectors (three male and four female) were deployed. To facilitate comfort and improve rapport, interviewers were matched to participants by sex—male interviewers conducted interviews with male participants and female interviewers with female participants. Field activities were overseen by staff from the Tulonge Afya project, who supervised sampling, respondent screening, adherence to the study protocol, and provided daily debriefings with data collectors. A structured, interviewer-administered questionnaire—translated into Kiswahili—was used for all interviews. Kiswahili was chosen as the language of administration due to its status as the national language and broad familiarity among participants. The questionnaire was adapted from several validated sources, including the Tanzania Demographic and Health Survey (TDHS), Tanzania HIV Impact Survey (THIS), and Tulonge Afya’s baseline, omnibus, and sentinel surveys. To ensure content validity and cultural appropriateness, the tool was piloted in Dar es Salaam, a region not included in the main study. The final version was digitized and deployed via password-protected Android tablets. Data were securely uploaded to an electronic database and organized using an English-language data dictionary. The questionnaire was organized into thematic sections. The first captured location and interview details (region, council, date, and interviewer ID). The second gathered socio-demographic information (age, sex, education, living situation, and number of children). The third section collected communication related information which addressed exposure to SBC interventions, including campaign and message recall, source, and intensity. Subsequent sections assessed behavioral and ideational factors (knowledge, risk perception, social dialogue, positive attitudes, perceived self-efficacy, and perceived norms) domains using a mix of Likert-scale and dichotomous questions. These included age of sexual debut, number of sexual partners, nature of relationships, condom use with partners, HIV testing behavior, discussions about sex, condom use, and HIV testing with different individuals in the community, awareness of condoms as a means of preventing HIV transmission, belief that any sexual encounter without contraception significantly increases the chance of pregnancy, belief that the consequences of becoming pregnant would adversely affect personal/future goals, perception of risk regarding HIV infection, belief in the safety of modern contraceptive methods, belief in the importance of access to sexual and reproductive health (SRH) services, belief in the effectiveness of condoms for preventing HIV and unwanted pregnancies), parental support in modern contraception to prevent pregnancy, parental support in HIV prevention, awareness of condom use among peers, involvement in health decision-making, confidence in the ability to refuse unprotected sex and confidence in the ability to persuade partner to use a condom in every sexual encounter. The average interview duration was approximately 50 minutes.

### Variables

The primary outcome variable was self-reported condom use during the most recent sexual encounter within the past six months. This was measured by asking participants whether they used a condom the last time they had sexual intercourse. Condom use at last sex is widely recognized as a reliable indicator of general condom use and is often used as a proxy for consistent condom use over time [26].

Independent variables were organized according to the study’s conceptual framework and included four domains: sociodemographic characteristics, behavioral factors, communication-related factors, and ideational (cognitive, emotional, social) factors. Communication-related factors captured self-reported exposure to SBC interventions, including recall of specific messages, the source or channel through which messages were encountered, and the intensity of message exposure within the six months preceding data collection. These were assessed through questions about what messages participants saw or heard, where they encountered them, and the number of exposures, six months preceding the study.

Ideational factors were developed based on constructs from the Tulonge Afya Youth SBC Strategy for HIV and Contraception [27]. These included domains such as knowledge, risk perception, social dialogue, positive attitudes, and social norms. Responses were collected using a four-point Likert scale (1 = Strongly agree, 2 = Agree, 3 = Disagree, 4 = Strongly disagree). Perceived self-efficacy was assessed separately using a three-point Likert scale (1 = Extremely confident, 2 = Moderately confident, 3 = Not at all confident). Cronbach’s alpha coefficients of 0.65 or higher were considered acceptable for internal consistency of the ideational scales [28,29].

For analysis, Likert-scale responses were recoded into binary variables. The most affirmative responses (e.g., “strongly agree” or “extremely confident”) were coded as 1 = Yes, indicating endorsement of the construct, while all other responses were coded as 0 = No.

### Statistical analysis

A total of 2,100 youth across the three regions were approached for participation, of whom 1,959 provided informed consent, resulting in a response rate of 93%. Reasons for non-participation included lack of time, discomfort with the sensitive nature of the questions, and unwillingness to participate without compensation. For the analysis, only respondents who reported being sexually active within the six months preceding the survey were included. Of the 1,959 participants who were approached, 1,265 (64.6%) met this criterion.

Data were analyzed using STATA version 15. Descriptive statistics were used to summarize sociodemographic, behavioral, communication related, and ideational factors among sexually active respondents. Categorical variables were described using frequencies and percentages, while continuous variables were summarized using means, medians, standard deviations, and ranges.

Bivariate and multivariate logistic regression analyses were conducted to assess associations between the outcome variable—condom use at last sexual intercourse, and the independent variables. Multicollinearity was assessed prior to model inclusion. Independent variables with p-values of <0.10 in a bivariate analysis were included in the multivariate model. A p-value of <0.05 was considered as statistically significant.

### Ethical considerations

Ethical approval for this study was obtained from the FHI 360 Protection of Human Subjects Committee (PHSC) and the Tanzania National Institute for Medical Research (NIMR). Informed consent procedures were tailored to the participants’ age groups and adhered to ethical guidelines for research involving human subjects. Three versions of informed consent forms were used: one for participants aged 18 years and older, one for minors aged 15–17 years, and one for parents or guardians providing consent for minors.

Data collectors followed a standardized recruitment script to explain the study’s purpose, procedures, and voluntary nature to potential participants. For minors, parental or guardian consent was obtained before the study was introduced to the youth. Interviews with minors were scheduled at a time and location that ensured both convenience and privacy. Up to three attempts were made to secure participant consent and complete the recruitment process. If a selected participant could not be reached after three attempts, another individual with similar characteristics (age, sex, and location) from the sampling frame was approached.

Only individuals who provided written informed consent (or assent in the case of minors, with corresponding parental consent) were included in the study. To ensure confidentiality, interviews were conducted in private settings away from other household members. Each interview was assigned a unique identification number, and no personally identifiable information, such as names or contact details, was collected or stored.

## Results

### Descriptive analysis

#### Sociodemographic characteristics.

The respondents’ age range was from 15 to 25 years, with a mean age of 20.61 years (SD 2.55). The sex ratio of respondents was approximately equal, with 51.0% identifying as male. Similarly, there was an even distribution of respondents across regions and places of residence, with 35.4% in Iringa and 51.9% in urban areas. Many of the sexually active respondents (65.3%) did not have children. About two-thirds (70.0%) reported living with their parents or elders (Table 1).

**Table 1:** Sociodemographic characteristics of sexually active respondents in the study.

Variable	Value	N = 1,265	%
Region	Tabora	384	30.4
	Iringa	448	35.4
	Mwanza	433	34.2
Residence	Urban	656	51.9
	Rural	609	48.1
Sex	Male	645	51.0
	Female	620	49.0
Age (years)	20 - 24	816	64.5
	15 - 19	449	35.5
Living with parent/elder	Yes	885	70.0
	No	380	30.0
Number of children	Two or more	95	7.5
	One	344	27.2
	None	826	65.3

#### Behavioral factors.

Nearly half (48.5%) indicated having sexual intercourse for the first time (sexual debut) between the ages of 15 and 17 years, with a mean age at sexual debut of 16.9 years (SD 2.33) (17.3 for females, 16.6 for males). Over three-fifths (64.7%) reported having one sexual partner in the last six months. More than three-quarters (84.0%) reported that their most recent sexual partner was a non-cohabiting partner. Less than three-quarters (70.1%) stated that they had tested for HIV within the six months prior to the study, while about two-thirds (64.6%) reported that their sexual partner had previously tested for HIV (Table 2).

**Table 2:** Behavioral factors among sexually active respondents in the study.

Variable	Value	N = 1,265	%
Age of sexual debut (years)	18+	520	41.1
	15 - 17	614	48.5
	Below 15	131	10.4
Number of sexual partners	2 or more	447	35.3
	One	818	64.7
Relationship to sexual partners	Non-cohabiting	1062	84.0
	Cohabiting	203	16.0
Tested for HIV in last 6 months	Yes	887	70.1
	No	378	29.9
Partner has tested for HIV before	Yes	817	64.6
	No	253	20.0
	Don’t know	195	15.4

#### Communication related factors.

Three-fifths (62.8%) of respondents recalled the SITETEREKI youth platform. More than half (55.7%) reported having seen a male condom demonstration. Two-fifths (39.7%) recalled hearing or seeing specific messaging about condom use, 42.5% remembered specific messaging on modern contraceptive use, 29.7% noted messaging on HIV testing and treatment adherence, and 8.1% recalled messaging on voluntary medical male circumcision within the six months leading up to the study. Additionally, 16.8% of respondents reported hearing or seeing specific messages about condom use from one source, while 11.9% reported three or more sources within that time frame. Regarding the sources of condom use messaging, 19.7% mentioned mass media, 12.0% printed materials, 2.1% social media, 29.8% interpersonal communication, and 8.0% mid media within the last six months before the study (Table 3).

**Table 3:** Communication related factors among sexually active respondents in the study.

Variable	Value	N = 1,265	%
Recalled the “SITETEREKI” youth platform	Yes	795	62.8
	No	470	37.2
Have seen a male condom demonstration	Yes	705	55.7
	No	560	44.3
Recalled specific messaging on condom use	Yes	502	39.7
	No	763	60.3
Recalled specific messaging on modern contraceptive use	Yes	538	42.5
	No	727	57.5
Recalled specific messaging on HIV testing and treatment adherence	Yes	343	27.1
	No	922	72.9
Recalled specific messaging on voluntary male medical circumcision	Yes	102	8.1
	No	1163	91.9
Heard/saw specific condom use messaging through mass media	Yes	249	19.7
	No	1016	80.3
Heard/Saw specific condom use messaging on print media	Yes	152	12.0
	No	1113	88.0
Heard/Saw specific condom use messaging through social media	Yes	27	2.1
	No	1238	97.9
Heard/saw specific condom use messaging through interpersonal communication	Yes	377	29.8
	No	888	70.2
Heard/Saw specific condom use messaging through mid media	Yes	101	8.0
	No	1164	92.0
Number of sources exposed for specific condom use messaging	3 or more	151	11.9
	2	139	11.0
	1	212	16.8
	0	763	60.3

#### Ideational factors.

A large majority (92.9%) knew that condoms can reduce the risk of HIV transmission. Additionally, 56.0% believed that an unplanned pregnancy might impact their future goals. One-third (30.9%) believed they were at risk of acquiring HIV, and half (50.0%) believed that sex without contraception could result in pregnancy. Four-fifths (82.3%) believed that having multiple sex partners puts someone at much higher risk of HIV and STIs. About three-fifths (62.8%) believed that condoms are effective in preventing unwanted pregnancies, while 58.9% believed that condoms effectively prevent HIV/STIs. Only one-tenth (10.6%) believed that peers in their communities use condoms. Two-thirds (67.9%) felt extremely confident in their ability to convince their partner to use a condom (Table 4).

**Table 4:** Ideational (cognitive and emotional) factors among sexually active respondents in the study.

Variable	Value	N = 1,265	%
Know condom use reduces the risk of HIV transmission	Yes	1175	92.9
	No	90	7.1
Believe unplanned pregnancy may affect their future goals	Yes	709	56.0
	No	556	44.0
Believe they are at risk of acquiring HIV	Yes	391	30.9
	No	874	69.1
Believe having multiple sex partners puts someone at much higher risk of HIV/STIs	Yes	1041	82.3
	No	224	17.7
Believe sex encounter without the use of any contraception will lead to pregnancy	Yes	632	50.0
	No	633	50.0
Believe condoms are effective in preventing HIV/STIs	Yes	745	58.9
	No	520	41.1
Believe condoms are effective in preventing unwanted pregnancy	Yes	794	62.8
	No	471	37.2
Believe modern contraceptive methods are safe for use	Yes	522	41.3
	No	743	58.7
Believe their peers are using condoms	Yes	134	10.6
	No	1131	89.4
Extremely confident to refuse sex without condom	Yes	658	52.0
	No	607	48.0
Extremely confident to convince partner to use condom	Yes	859	67.9
	No	406	32.1

More than half (58.3%) of respondents made health decisions independently. About one-tenth (8.9%) of the participants reported having discussed sex with a parent or elder, while half (50.0%) indicated discussing sex with their partner. Only 4.0% of participants reported discussing sex with a facility-based health provider. Approximately one-third (29.9%) of participants reported discussing condoms with a parent or elder, whereas 4.3% reported discussing condom use with their partner, and just 1.4% spoke with a facility-based health provider about condom use. More than one-third (39.1%) talked about HIV counseling and testing (HCT) with a partner, while 7.7% discussed it with a parent or elder, and 5.9% did so with a health provider. More than one-third (37.2%) believed that parents support HIV prevention. In comparison, only a quarter (27.3%) believed that parents support the use of modern contraceptive methods to prevent unplanned pregnancies (Table 5).

**Table 5:** Ideational (social) factors among sexually active respondents in the study.

Variable	Value	N = 1,265	%
Involvement in decision making of health	Joint with partner	84	6.6
	Partner	80	6.3
	Self	738	58.3
	Parent/Relative	363	28.7
Dialogue about sex with parent/elder	Yes	113	8.9
	No	1152	91.1
Dialogue about sex with partner	Yes	632	50.0
	No	633	50.0
Dialogue about sex with a facility health provider	Yes	51	4.0
	No	1214	96.0
Dialogue about condoms with parent/elder	Yes	378	29.9
	No	887	70.1
Dialogue about condoms with partner	Yes	55	4.3
	No	1210	95.7
Dialogue about condoms with a facility health provider	Yes	18	1.4
	No	1247	98.6
Dialogue about HIV counseling and testing with parent/elder	Yes	98	7.7
	No	1167	92.3
Dialogue about HIV counseling and testing with partner	Yes	495	39.1
	No	770	60.9
Dialogue about HIV counseling and testing with a facility health provider	Yes	75	5.9
	No	1190	94.1
Believe teenage pregnancy is taken seriously in their communities	Yes	816	64.5
	No	449	35.5
Believe it is important for youth to have access to SRH information and services	Yes	569	45.0
	No	696	55.0
Believe parents are supportive in HIV prevention	Yes	471	37.2
	No	794	62.8
Believe parents support the use of modern contraceptive methods to prevent pregnancy	Yes	345	27.3
	No	920	72.7
Believe it is not the woman’s responsibility to avoid pregnancy	Yes	460	36.4
	No	805	64.6

### Bivariate analysis

Overall, fewer than half (46.9%) of sexually active respondents reported using condoms during their last sexual encounter in the six months preceding the study (95% CI: 44.0–50.0). The relationships of sociodemographic, behavioral, communication-related, and ideational factors with condom use among sexually active youth are presented in Tables 6–9, respectively. Among sociodemographic factors, residing in Tabora region (OR = 1.702; 95% CI: 1.290–2.246; p < 0.001), living with parents/elders (OR = 1.277; 95% CI: 1.003–1.628; p = 0.048), having no children (OR = 1.903; 95% CI: 1.217–2.975; p = 0.005), and having one child (OR = 1.615; 95% CI: 1.003–2.598; p = 0.048), were significantly associated with condom use. Additionally, behavioral factors, including delayed sexual debut (OR = 1.482; 95% CI: 1.005–2.184; p = 0.047), having multiple sexual partners (OR = 1.506; 95% CI: 1.195–1.899; p = 0.001), respondent’s HIV testing behavior (OR = 1.493; 95% CI: 1.169–1.906; p = 0.001), and partner’s HIV testing behavior (OR = 1.951; 95% CI: 1.410–2.697; p < 0.001), were significantly associated with condom use (Table 6). On the other hand, there was no association between condom use and other factors such as the age of the respondent and the nature of the relationship to sexual partners.

**Table 6:** Bivariate analysis of sociodemographic characteristics and behavioral factors for condom use among sexually active youth.

Variable	Sexually active youth (N = 1,265) n (%)	Condom use (N = 593) n (%)	OR	95% CI	*P-value*
**Region**					
Tabora	384	221 (57.6)	1.702	1.290–2.246	<0.001*
Iringa	448	180 (40.2)	0.843	0.645–1.102	0.211
Mwanza	433	192 (44.3)	Ref		
**Age (years)**					
20 - 24	816	398 (48.8)	1.240	0.983–1.563	0.069
15 - 19	449	195 (43.4)	Ref		
**Living with parent/elder**					
Yes	885	431 (48.7)	1.277	1.003–1.628	0.048*
No	380	162 (42.6)	Ref		
**Number of children**					
None	826	406 (49.2)	1.903	1.217–2.975	0.005*
One	344	155 (45.1)	1.615	1.003–2.598	0.048*
Two or more	95	32 (33.7)	Ref		
**Age of sexual debut (years)**					
18+	520	265 (51.0)	1.482	1.005–2.184	0.047*
15 - 17	614	274 (44.6)	1.149	0.784–1.685	0.476
Below 15	131	54 (41.2)	Ref		
**Number of sexual partners**					
2 or more	447	239 (53.5)	1.506	1.195–1.899	0.001*
One	818	354 (43.3)	Ref		
**Relationship to sexual partners**					
Non-cohabiting	1062	507 (47.7)	1.243	0.918–1.683	0.160
Cohabiting	203	86 (42.4)	Ref		
**Tested for HIV in last 6 months**					
Yes	887	442 (49.8)	1.493	1.169–1.906	0.001*
No	378	151 (39.9)	Ref		
**Partner has tested for HIV before**					
Yes	817	422 (51.7)	1.951	1.410–2.697	<0.001*
No	253	102 (40.3)	1.233	0.838–1.815	0.287
Don’t know	195	69 (35.4)	Ref		

*statistically significant 95% level of confidence at a p-value below 0.05 using a two-tailed t-test. CI = Confidence interval; OR = Odds ratio; Ref = Reference.

**Table 7:** Bivariate analysis of communication related factors for condom use among sexually active youth.

Variable	Sexually active youth (N = 1,265) n (%)	Condom use (N = 593) n (%)	OR	95% CI	*P-value*
**Recalled the youth platform “SITETEREKI”**					
Yes	795	401 (50.4)	1.474	1.170–1.856	0.001*
No	470	192 (40.9)	Ref		
**Have seen a male condom demonstration**					
Yes	705	358 (50.8)	1.427	1.141–1.784	0.002*
No	560	235 (42.0)	Ref		
**Recalled specific messaging on condom use**					
Yes	502	264 (52.6)	1.463	1.167–1.834	0.001*
No	763	329 (43.1)	Ref		
**Recalled specific messaging on modern contraceptive use**					
Yes	538	279 (51.9)	1.417	1.133–1.772	0.002*
No	727	314 (43.2)	Ref		
**Recalled specific messaging on voluntary male medical circumcision**					
Yes	102	61 (59.8)	1.765	1.168–2.665	0.007*
No	1163	523 (45.7)	Ref		
**Heard/saw specific condom use messaging through mass media**					
Yes	249	136 (54.6)	1.472	1.114–1.945	0.006*
No	1016	457 (45.0)	Ref		
**Heard/Saw specific condom use messaging through social media**					
Yes	27	19 (70.4)	2.747	1.194–6.323	0.017*
No	1238	574 (46.4)	Ref		
**Heard/Saw specific condom use messaging on print media**					
Yes	152	75 (49.3)	1.119	0.797–1.570	0.516
No	1113	518 (46.5)	Ref		
**Heard/saw specific condom use messaging through interpersonal communication**					
Yes	377	203 (53.8)	1.490	1.169–1.898	0.001*
No	888	390 (43.9)	Ref		
**Heard/Saw specific condom use messaging through mid media**					
Yes	101	57 (56.4)	1.518	1.007–2.287	0.046*
No	1164	536 (46.0)	Ref		
**Number of sources exposed for specific condom use messaging**					
3 or more	151	87 (57.2)	1.793	1.260–2.553	0.001*
2	139	70 (50.4)	1.338	0.932–1.922	0.115
1	212	107 (50.5)	1.344	0.991–1.824	0.057
0	763	329 (43.1)	Ref		

**Note:** * statistically significant 95% level of confidence at a p-value below 0.05 using a two-tailed t-test. CI = Confidence interval; OR = Odds ratio; Ref = Reference.

**Table 8:** Bivariate analysis of ideational (cognitive and emotional) factors for condom use among sexually active youth.

Variable	Sexually active youth (N = 1,265) n (%)	Condom use (N = 593) n (%)	OR	95% CI	*P-value*
**Know condom use reduces the risk of HIV transmission**					
Yes	1175	565 (48.1)	2.051	1.294–3.251	0.002*
No	90	28 (31.1)	Ref		
**Believe unplanned pregnancy may affect their future goals**					
Yes	709	361 (50.9)	1.449	1.158–1.812	0.001*
No	556	232 (41.7)	Ref		
**Believe they are at risk of acquiring HIV**					
Yes	391	172 (44.0)	0.845	0.665–1.074	0.169
No	874	421 (48.2)	Ref		
**Believe having multiple sex partners puts someone at much higher risk of HIV/STI**					
Yes	1041	499 (47.9)	1.273	0.951–1.705	0.105
No	224	94 (42.0)	Ref		
**Believe sex encounter without the use of any contraception will lead to pregnancy**					
Yes	632	325 (51.4)	1.442	1.155–1.800	0.001*
No	633	268 (42.3)	Ref		
**Believe condoms are effective in preventing HIV/STIs**					
Yes	745	373 (50.1)	1.367	1.091–1.713	0.007*
No	520	220 (42.3)	Ref		
**Believe condoms are effective in preventing unwanted pregnancy**					
Yes	794	397 (50.0)	1.403	1.115–1.766	0.004*
No	471	196 (41.6)	Ref		
**Extremely confident to refuse sex without condom**					
Yes	658	334 (50.8)	1.385	1.109–1.729	0.004*
No	607	259 (42.7)	Ref		
**Extremely confident to convince partner to use condom**					
Yes	859	448 (52.2)	1.962	1.538–2.502	<0.001*
No	406	145 (35.7)	Ref		
**Believe modern contraceptive methods are safe for use**					
Yes	522	263 (50.4)	1.271	1.015–1.591	0.036*
No	743	330 (44.4)	Ref		

*statistically significant 95% level of confidence at a p-value below 0.05 using a two-tailed t-test. CI = Confidence interval; OR = Odds ratio; Ref = Reference.

**Table 9:** Bivariate analysis of ideational (social) factors for condom use among sexually active youth.

Variable	Sexually active youth (N = 1,265) n (%)	Condom use (N = 593) n (%)	OR	95% CI	*P-value*
**Involvement in decision making of health**					
Joint with partner	84	41 (48.8)	1.872	0.996–3.517	0.051
Self	738	355 (48.1)	1.819	1.120–2.956	0.016*
Parent/Relative	363	170 (46.8)	1.729	1.041–2.871	0.034*
Partner	80	27 (33.8)	Ref		
**Dialogue about sex with partner**					
Yes	632	315 (49.8)	1.269	1.017–1.583	0.035*
No	633	278 (43.9)	Ref		
**Dialogue about condoms with parent/elder**					
Yes	378	235 (62.2)	2.428	1.896–3.110	<0.001*
No	887	358 (40.4)	Ref		
**Dialogue about condoms with partner**					
Yes	55	35 (63.6)	2.045	1.167–3.583	0.012*
No	1210	558 (46.1)	Ref		
**Dialogue about HIV testing and counseling with partner**					
Yes	495	263 (53.9)	1.511	1.204–1.897	<0.001*
No	770	330 (42.9)	Ref		
**Dialogue about HIV testing and counseling with a facility health provider**					
Yes	75	44 (58.7)	1.657	1.032–2.661	0.035*
No	1190	549 (46.1)	Ref		
**Believe teenage pregnancy is taken seriously in their communities**					
Yes	816	395 (48.4)	1.189	0.944–1.499	0.142
No	449	198 (44.1)	Ref		
**Believe parents are supportive in HIV prevention**					
Yes	471	253 (53.7)	1.550	1.232–1.949	<0.001*
No	794	340 (42.8)	Ref		
**Believe parents support the use of modern contraceptive methods to prevent pregnancy**					
Yes	345	176 (51.0)	1.256	0.980–1.609	0.071
No	920	417 (45.3)	Ref		
**Believe it is important for youth to have access to SRH information and services**					
Yes	569	283 (49.7)	1.232	0.987–1.538	0.065
No	696	310 (44.5)	Ref		

*statistically significant 95% level of confidence at a p-value below 0.05 using a two-tailed t-test. CI = Confidence interval; OR = Odds ratio; Ref = Reference.

Among communication related factors (Table 7), condom use was significantly associated with exposure, campaign and message recall. Recalling the SITETEREKI platform (OR = 1.474; 95% CI: 1.170–1.856; p = 0.001), condom use messaging (OR = 1.463; 95% CI: 1.167–1.834; p = 0.001), modern contraception messaging (OR = 1.417; 95% CI: 1.133–1.772; p = 0.002), and voluntary medical male circumcision messaging (OR = 1.765; 95% CI: 1.168–2.665; p = 0.007) were significantly associated with condom use. Additionally, the findings also indicate that condom use was significantly associated with the source and intensity of condom messaging exposure. The likelihood of condom use among sexually active youth who heard or saw specific condom use messaging through demonstrations (OR = 1.427; 95% CI: 1.141–1.784; p = 0.002), mass media (OR = 1.472; 95% CI: 1.114–1.945; p = 0.006), social media (OR = 2.747; 95% CI: 1.194–6.323; p = 0.017), interpersonal communication (OR = 1.490; 95% CI: 1.169–1.898; p = 0.001), and mid media (OR = 1.518; 95% CI: 1.007–2.287; p = 0.046) was higher than those who did not. Being exposed to three or more sources conveying condom behavior messaging (OR = 1.793; 95% CI: 1.260–2.553; p = 0.001) was also significantly associated with condom use. However, there was no association between condom use and exposure to condom messaging through print media.

Furthermore, condom use was significantly associated with some cognitive (knowledge, risk perception, positive attitudes, perceived norms) and emotional ideational (perceived self-efficacy) factors (Table 8). Specifically, condom use was significantly associated with respondents who knew condoms reduce the risk of HIV transmission (OR = 2.051; 95% CI: 1.294–3.251; p = 0.002), believed an unplanned pregnancy may affect their future goals (OR = 1.449; 95% CI: 1.158–1.812; p = 0.001), believed that sex without the use of any contraception would lead to pregnancy (OR = 1.442; 95% CI: 1.155–1.800; p = 0.001), believed condoms were effective in preventing HIV/STIs (OR = 1.367; 95% CI: 1.091–1.713; p = 0.007) and unwanted pregnancy (OR = 1.403; 95% CI: 1.115–1.766; p = 0.004) respectively, and believed modern contraceptive methods were safe for use (OR = 1.271; 95% CI: 1.015–1.591; p = 0.036). There was also a significant association between youth who were extremely confident in refusing sex without a condom (OR = 1.385; 95% CI: 1.109–1.729; p = 0.004) and convincing their partner to use a condom (OR = 1.962; 95% CI: 1.538–2.502; p < 0.001) respectively.

On the other hand, condom use was significantly associated with a few social ideational factors, including health decision making involvement, social dialogue, and perceived parental support (Table 9). Specifically, condom use was significantly associated with making health decisions independently (OR = 1.819; 95% CI: 1.120–2.956; p = 0.016) and by parents or relatives (OR = 1.729; 95% CI: 1.041–2.871; p = 0.034). Additionally, the likelihood of condom use was higher among respondents who had discussed sex with a partner (OR = 1.269; 95% CI: 1.017–1.583; p = 0.035), discussed condoms with parents (OR = 2.428; 95% CI: 1.896–3.110; p < 0.001), discussed condoms with a partner (OR = 2.045; 95% CI: 1.167–3.583; p = 0.012), discussed HIV testing and counseling with a partner (OR = 1.511; 95% CI: 1.204–1.897; p < 0.001), and discussed HIV testing and counseling with a facility health provider (OR = 1.657; 95% CI: 1.032–2.661; p = 0.035). There was a significant association between youth who believed that parents were supportive of HIV prevention (OR = 1.550; 95% CI: 1.232–1.949; p < 0.001) and condom use.

### Multivariate analysis

After fitting into the multivariable logistic regression model (Appendix 1), the results indicate that sociodemographic characteristics (region and number of children), behavioral factors (age of sexual debut, number of sexual partners, and partners’ HIV testing behavior), emotional ideational factors (perceived self-efficacy to convince partner on condom use), and social ideational factors (parental discussions about condom use, and perceived parental support in HIV prevention) remained significantly associated with condom use among sexually active youth (Table 10). However, none of the communication related and cognitive ideational factors were found to be significantly associated with condom use in this analysis.

**Table 10:** Multivariate logistic regression model showing factors associated with condom use among sexually active youths.

Variable	Condom use (n = 593)	AOR	95% CI		*P-value*
			Lower	Upper	
**Sociodemographic Characteristics**					
**Region**					
Tabora	221	1.609	1.155	2.241	0.005*
Iringa	180	0.986	0.717	1.356	0.932
Mwanza	192	Ref			
**Number of children**					
None	406	1.844	1.095	3.106	0.021*
One	155	1.522	0.887	2.609	0.127
Two or more	32	Ref			
**Behavioral Factors**					
**Age of sexual debut (years)**					
18+	265	1.591	1.027	2.464	0.038*
15 - 17	274	1.202	0.793	1.824	0.386
Below 15	54	Ref			
**Number of sexual partners**					
2 or more	239	1.667	1.280	2.171	<0.001*
One	354	Ref			
**Partner has tested for HIV**					
Yes	422	1.671	1.138	2.455	0.009*
No	102	1.257	0.823	1.920	0.289
Don’t Know	69	Ref			
**Communication Related Factors**					
**Recalled specific messaging on voluntary male medical circumcision**					
Yes	61	1.593	0.977	2.595	0.062
No	523	Ref			
**Heard/Saw specific condom use messaging through social media**					
Yes	19	2.367	0.916	6.115	0.075
No	574	Ref			
**Ideational Factors**					
**Involvement in decision making of health**					
Joint with partner	41	1.820	0.909	3.644	0.091
Self	355	1.527	0.891	2.616	0.123
Parent/Relative	170	1.647	0.935	2.901	0.084
Partner	27	Ref			
**Dialogue about condoms with parent/elder**					
Yes	235	1.902	1.430	2.532	<0.001*
No	358	Ref			
**Dialogue about condoms with partner**					
Yes	35	1.803	0.962	3.380	0.066
No	558	Ref			
**Know condom use reduces the risk of HIV transmission**					
Yes	565	1.525	0.916	2.539	0.104
No	28	Ref			
**Extremely confident to convince partner to use condom**					
Yes	448	1.653	1.233	2.215	0.001*
No	145	Ref			
**Believe parents are supportive in HIV prevention**					
Yes	253	1.585	1.200	2.095	0.001*
No	340	Ref			
**Believe it is important for youth to have access to SRH information and services**					
Yes	263	0.777	0.565	1.069	0.121
No	330	Ref			

*statistically significant 95% level of confidence at a p-value below 0.05 using a two-tailed t-test. CI = Confidence interval; AOR = Adjusted odds ratio; Ref = Reference.

Youth in Tabora were more likely to use condoms compared to those in Iringa and Mwanza (AOR = 1.609; 95% CI: 1.155–2.241; p = 0.005). Youth without children were more likely to use condoms than those with children (AOR = 1.844; 95% CI: 1.095–3.106; p = 0.021).

Youth who began engaging in sexual activity at the age of 18 years or older were more likely to use condoms compared to those who started sexual activity before age 18 (AOR = 1.591; 95% CI: 1.027–2.464; p = 0.038). Respondents with two or more sexual partners were more likely to use condoms compared to those with only one partner (AOR = 1.667; 95% CI: 1.280–2.171; p < 0.001). Similarly, respondents whose sexual partner tested for HIV were more likely to use condoms than those whose partners did not test or were uncertain (AOR = 1.671; 95% CI: 1.138–2.455; p = 0.009).

Youth who discussed condom use with their parents or elders were more likely to use condoms compared to those who did not (AOR = 1.902; 95% CI: 1.430–2.532; p < 0.001). Additionally, respondents who were very confident in proposing condom use to their sexual partners were more likely to use condoms than those who were not very confident (AOR = 1.653; 95% CI: 1.233–2.215; p = 0.001), while respondents who believed that their parents supported HIV prevention were more likely to use condoms than those who did not (AOR = 1.585; 95% CI: 1.200–2.095; p = 0.001).

## Discussion

This study is significant because it describes condom use behavior among sexually active youth within SBC intervention communities in Tanzania. The study assessed condom use at last sexual activity and the factors associated with this behavior among sexually active youth in these settings. The main principle behind the conceptual framework is that the adoption of a recommended behavior is more likely to occur in a specific setting when individuals have acquired sufficient knowledge, maintain positive attitudes, engage in discussions with others, receive the necessary support, experience positive emotions, and possess confidence in their ability to act [17,21].

The results of the descriptive analysis showed that 46.9% of sexually active youth reported using a condom at last sex (47.0% among males and 46.8% among females). This finding is similar to a study by Kalolo et al., which reported that 50.3% of sexually active adolescents aged 14–19 years in Tanzania used a condom at their last sexual encounter [20]. However, this finding is higher compared to other previous studies reported in Tanzania, Nigeria, Ethiopia, and Malawi, where condom use during the last sexual encounter was reported at 32.0%, 42.3%, 35.2%, and 27.1%, respectively [3,30–32] which suggests encouraging progress in condom use uptake among youth. These findings indicate that collaborative efforts between the Government of Tanzania and development partners in preventing HIV, STIs, and unintended pregnancies among youth are essential for improved coordination and coverage of comprehensive condom programming [3,16,33]. Future programs should continually employ holistic approaches, integrating multisectoral and complementary efforts to ensure high-quality, sustainable, and impactful interventions.

The results from the multivariable analysis demonstrate that several sociodemographic factors (region and number of children), behavioral factors (age of sexual debut, number of sexual partners, and partners’ HIV testing behavior), emotional ideational factors (perceived self-efficacy to convince partner on condom use) and social ideational factors (parental discussions about condom use, and perceived parental support for HIV prevention) were significantly associated with condom use among sexually active youth within SBC intervention communities.

Youth residing in Tabora were more likely to use condoms compared to those in Iringa and Mwanza, echoing findings from Malawi that regional differences can influence condom use among male and female youth [32]. One possible explanation for this association could be that during the time of the study, the project focused on implementing intensified community-level SBC activities using regional mass media, mid media, and interpersonal communication approaches in geographical areas with higher unmet contraception needs, including Tabora. This involved collaborating with implementing partners who support the provision of comprehensive health service delivery for preventative and curative services in family planning, and HIV/AIDS within those regions. Hence, future programs should consider strong local partnerships when aligning SBC activities with service delivery mechanisms to achieve significant gains in settings with contextual health needs.

Youth without children also had higher odds of condom use, which is consistent with studies from Cameroon [34]. Youth with two or more sexual partners were more likely to use condoms, consistent with studies from Ethiopia, Nepal, Botswana, and Haiti [31,35–37]. These results indicate that condom use may be driven by perceived susceptibility to unwanted pregnancies, HIV infection, and other STIs through risky behaviors among sexually active youth. Future programs should focus on showing how failing to use condoms could increase the chance of unintended pregnancies among sexually active youth, which could derail their life plans, as well as the benefits of condom use as dual protection for HIV and unplanned pregnancy [3,27,38].

Conversely, delayed sexual debut (18 + years) was associated with higher odds of condom use, aligning with studies from Taiwan and Eswatini [39,40]. This result could be attributed to maturity at sexual debut and adequate knowledge and skills to use condoms during intercourse [41]. Future programs should tailor interventions to reach younger adolescents to empower them with comprehensive knowledge and skills on safe sex practices, including condom use and delayed sex initiation.

Additionally, partner HIV testing was a significant predictor of condom use, consistent with findings from South Africa, which found condom use was associated with knowledge of partner’s HIV status [42]. This indicates that higher health consciousness and risk perception among sexual partners may lead to more responsible behaviors such as condom use. It also suggests that awareness and communication about HIV status may play a critical role in shaping safer sexual practices [42]. This can be explained by the fact that the majority (70.1%) of sexually active youth reported having tested for HIV within the last 6 months before the study. Therefore, future programs should incorporate messaging that emphasizes the importance of testing if at risk, knowing a sexual partner’s HIV status, and the benefits of mutual protection among youth.

Parental dialogue about condom use and perceived parental support for HIV prevention emerged as significant ideational (social) predictors of condom use. These results are supported by prior studies reported by Atienzo et al., Hadley et al., Namisi et al., Tarkang et al., and James et al. [43–47]. This indicates that parental support and communication may have a significant impact on youth sexual behavior, which helps prevent unwanted pregnancy and STIs, including HIV. Additionally, parental communication and support can enhance emotional and social connectedness, motivating youth to meet their parents’ expectations and likely reducing engagement in risky sexual behavior that could lead to unplanned pregnancy or STIs [47]. Future program interventions should integrate parents as key stakeholders to equip them with the necessary skills and tools to foster open and supportive dialogue about sexual reproductive health with youth, including condom use and safer sex practices.

Furthermore, perceived self-efficacy to convince partner on condom use was significantly associated with condom use, echoing findings by Carmack et al., who reported that youth who talked to their partner about using a condom before engaging in sexual activity were more likely to practice consistent condom use [48]. This indicates that supportive norms surrounding discussions about sexual and reproductive health can enhance condom negotiation among youth and lead to desired outcomes [27,48,49]. Future programs should focus on advancing interventions that promote condom communication and equip youth with the necessary tools and skills to express their preferences before sexual encounters.

Interestingly, none of the communication related factors were significantly associated with condom use among youth in the multivariate analysis of this study. This finding contrasts with several studies that have highlighted the influence of health communication on condom use in low- and middle-income countries [50]. Our best estimate is that the youth who were exposed to condom messaging through social media and recalled VMMC messaging have increased odds of condom use. However, our estimates are imprecise, and we cannot rule out the possibility that communication exposure may be associated with condom use among sexually active youth. Additionally, the literature indicates that communication can directly influence cognitive, emotional, and social factors of ideation individually and collectively, as these factors are interdependent and often occur simultaneously [51]. Hence, future research should consider using different study designs to examine the effects and relationships (both indirect and mediating) associated with communication related factors and significant factors identified in this study [52].

## Strength and limitations

The strength of this study is that it provides valuable insights into the theoretical framework adapted, and important lessons can be learned. It is a useful case study within the Tanzanian context and SBC field because it has comprehensively described various factors influencing health behavior specific to a high-risk population. However, some limitations in this study should be noted. The study used a cross-sectional design, which limits ascertaining causal inferences and drawing causal relationship conclusions between variables. The study could not accommodate larger sample sizes than 2,100 due to budgetary limitations and the resources (time, financial) needed for data collection and analysis. The study responses depended on participants’ self-reported condom use behavior and other aspects of their sexual activity. Hence, recall bias may have affected this study, which is common in cross-sectional study data. This may have affected the ability of study respondents to recall whether a condom was used or not during the last sexual encounter, the age of sexual debut, recall of health messaging, intensity, and source of message exposure, which could contribute to under-reporting or over-reporting of the study findings. Social desirability could have also affected the study, which is relatively common in studies associated with sexual and reproductive health among youth. Study respondents may have felt the desire to appear risk-averse, non-promiscuous, and favorable to condom use, which could lead to over-reporting of the focused behavior. The convenience sampling of wards within intervention councils might have introduced some bias, which may explain the large proportion of youth reporting using condoms at last sex compared to other studies with similar populations. Despite the conceptual framework of this study being adapted from the ideational model of strategic communication and behavior change, some of the constructs of this theory, including environmental context factors such as condom access and availability, health provider bias, the burden of disease/public health issue, and other ideational factors like personal advocacy, pleasure/satisfaction, and self-image were not assessed in this study. Future studies could expand on these dimensions to provide a more comprehensive understanding of the factors associated with condom use among youth.

## Conclusion

This study identified a range of sociodemographic, behavioral, and ideational (emotional and social) factors associated with condom use among sexually active youth within SBC intervention communities in Tanzania. The findings enhance understanding of the multiple factors shaping condom use behavior and provide evidence to inform the design and implementation of targeted sexual and reproductive health interventions. The results highlight the importance of tailoring program strategies to the specific needs and contexts of young people to improve the uptake of protective behaviors. Future programs should integrate contextually relevant and youth-centered approaches that address both individual and structural barriers to condom use. Additionally, further research is needed to investigate the underlying effects and relationships among the identified factors to strengthen the evidence base for effective intervention strategies.

## Supporting information

S1 Appendix Variables included in the final multivariable logistic regression model. (DOCX)

S1 File Inclusivity in global research questionnaire. (DOCX)
