# Supplementary material for: Determinants of condom use among sexually active youth in SBC intervention communities in Tanzania: A cross-sectional study
Source: PLoS One. 2025 Jul 2;20(7):e0326878. doi: 10.1371/journal.pone.0326878 (PMC12221024; doi:10.1371/journal.pone.0326878)
Supplement: S1 Appendix — (DOCX) [file pone.0326878.s001.docx]

**Appendix 1. Variables included in the final multivariable logistic regression model**

| **Variable** | **AOR** | **95% CI** | | **P-value** |
| --- | --- | --- | --- | --- |
|  |  | **Lower** | **Upper** |  |
| **Region** | | | | |
| Iringa | 0.986 | 0.717 | 1.356 | 0.932 |
| Mwanza | Ref |  |  |  |
| Tabora | 1.609 | 1.155 | 2.241 | 0.005* |
| **Age (years)** | | | | |
| 15 - 19 | Ref |  |  |  |
| 20 - 24 | 1.190 | 0.895 | 1.581 | 0.231 |
| **Living with parent/elder** | | | | |
| No | Ref |  |  |  |
| Yes | 1.175 | 0.890 | 1.552 | 0.255 |
| **Number of children** | | | | |
| None | 1.844 | 1.095 | 3.106 | 0.021* |
| One | 1.522 | 0.887 | 2.609 | 0.127 |
| Two or more | Ref |  |  |  |
| **Age of sexual debut (years)** | | | | |
| Below 15 | Ref |  |  |  |
| 15 - 17 | 1.202 | 0.793 | 1.824 | 0.386 |
| 18+ | 1.591 | 1.027 | 2.464 | 0.038* |
| **Number of sexual partners** | | | | |
| One | Ref |  |  |  |
| 2 or more | 1.667 | 1.280 | 2.171 | 0.000* |
| **Tested for HIV in last 6 months** | | | | |
| No | Ref |  |  |  |
| Yes | 1.050 | 0.762 | 1.448 | 0.765 |
| **Partner has tested for HIV before** | | | | |
| No | 1.257 | 0.823 | 1.920 | 0.289 |
| Yes | 1.671 | 1.138 | 2.455 | 0.009* |
| Dont know | Ref |  |  |  |
| **Have seen a male condom demonstration** | | | | |
| No | Ref |  |  |  |
| Yes | 1.011 | 0.782 | 1.308 | 0.934 |
| **Recalled the “SITETEREKI” youth platform** | | | | |
| No | Ref |  |  |  |
| Yes | 1.120 | 0.850 | 1.475 | 0.419 |
| **Recalled specific messaging on condom use** | | | | |
| No | Ref |  |  |  |
| Yes | 0.702 | 0.243 | 2.031 | 0.514 |
| **Recalled specific messaging on modern contraceptive use** | | | | |
| No | Ref |  |  |  |
| Yes | 1.053 | 0.790 | 1.403 | 0.724 |
| **Recalled specific messaging on voluntary male medical circumcision** | | | | |
| No | Ref |  |  |  |
| Yes | 1.593 | 0.977 | 2.595 | 0.062 |
| **Heard/saw specific condom use messaging through mass media** | | | | |
| No | Ref |  |  |  |
| Yes | 1.193 | 0.698 | 2.038 | 0.518 |
| **Heard/Saw specific condom use messaging through social media** | | | | |
| No | Ref |  |  |  |
| Yes | 2.367 | 0.916 | 6.115 | 0.075 |
| **Heard/Saw specific condom use messaging through mid media** | | | | |
| No | Ref |  |  |  |
| Yes | 1.270 | 0.714 | 2.257 | 0.416 |
| **Heard/saw specific condom use messaging through interpersonal communication** | | | | |
| No | Ref |  |  |  |
| Yes | 1.204 | 0.666 | 2.175 | 0.539 |
| **Number of sources exposed for specific condom use messaging** | | | | |
| 0 | Ref |  |  |  |
| 1 | 1.302 | 0.630 | 2.692 | 0.476 |
| 2 | 0.997 | 0.553 | 1.799 | 0.993 |
| 3 or more | 1.000 | 0.000 | 1.000 | 0.999 |
| **Involvement in decision making of health** | | | | |
| Parent/Relative | 1.647 | 0.935 | 2.901 | 0.084 |
| Self | 1.527 | 0.891 | 2.616 | 0.123 |
| Partner | Ref |  |  |  |
| Joint with partner | 1.820 | 0.909 | 3.644 | 0.091 |
| **Dialogue about sex with partner** | | | | |
| No | Ref |  |  |  |
| Yes | 0.995 | 0.765 | 1.292 | 0.968 |
| **Dialogue about condoms with parent/elder** | | | | |
| No | Ref |  |  |  |
| Yes | 1.902 | 1.430 | 2.532 | 0.000* |
| **Dialogue about condoms with partner** | | | | |
| No | Ref |  |  |  |
| Yes | 1.803 | 0.962 | 3.380 | 0.066 |
| **Dialogue about HIV counseling and testing with partner** | | | | |
| No | Ref |  |  |  |
| Yes | 0.876 | 0.655 | 1.170 | 0.370 |
| **Dialogue about HIV counseling and testing with a facility health provider** | | | | |
| No | Ref |  |  |  |
| Yes | 1.201 | 0.708 | 2.036 | 0.497 |
| **Know condom use reduces the risk of HIV transmission** | | | | |
| No | Ref |  |  |  |
| Yes | 1.525 | 0.916 | 2.539 | 0.104 |
| **Believe unplanned pregnancy may affect their future goals** | | | | |
| No | Ref |  |  |  |
| Yes | 1.120 | 0.820 | 1.529 | 0.476 |
| **Believe sex encounter without the use of any contraception will lead to pregnancy** | | | | |
| No | Ref |  |  |  |
| Yes | 1.131 | 0.835 | 1.530 | 0.427 |
| **Believe condoms are effective in preventing HIV/STIs** | | | | |
| No | Ref |  |  |  |
| Yes | 0.838 | 0.563 | 1.248 | 0.385 |
| **Believe condoms are effective in preventing unwanted pregnancy** | | | | |
| No | Ref |  |  |  |
| Yes | 1.065 | 0.702 | 1.615 | 0.768 |
| **Extremely confident to refuse sex without condom** | | | | |
| No | Ref |  |  |  |
| Yes | 1.025 | 0.786 | 1.336 | 0.857 |
| **Extremely confident to convince partner to use condom** | | | | |
| No | Ref |  |  |  |
| Yes | 1.653 | 1.233 | 2.215 | 0.001* |
| **Believe parents are supportive in HIV prevention** | | | | |
| No | Ref |  |  |  |
| Yes | 1.585 | 1.200 | 2.095 | 0.001* |
| **Believe parents support the use of modern contraceptive methods to prevent pregnancy** | | | | |
| No | Ref |  |  |  |
| Yes | 1.070 | 0.764 | 1.500 | 0.693 |
| **Believe it is important for youth to have access to SRH information and services** | | | | |
| No | Ref |  |  |  |
| Yes | 0.777 | 0.565 | 1.069 | 0.121 |
| **Believe modern contraceptive methods are safe for use** | | | | |
| No | Ref |  |  |  |
| Yes | 0.908 | 0.648 | 1.273 | 0.577 |

**Note:** * statistically significant 95% level of confidence at a p-value below 0.05 using a two-tailed t-test. CI = Confidence interval; AOR = Adjusted odds ratio; Ref = Reference
